# Photodegradation Behavior of Nanosilica-Filled PMMA Composite: Cooperative Effect of Mixed Solvents and Interfacial Functional Groups

**DOI:** 10.3390/polym17162241

**Published:** 2025-08-19

**Authors:** Zhiping Xu, Liangchen Li, Ying Liu, Rui Yang

**Affiliations:** Department of Chemical Engineering, Tsinghua University, Beijing 100084, China; xuzp15@163.com (Z.X.); llc23@mails.tsinghua.edu.cn (L.L.); lyy-76@163.com (Y.L.)

**Keywords:** interfacial interaction, photodegradation of PMMA, solvent reactions, Py-GC-MS, aging process

## Abstract

Poly(methyl methacrylate) (PMMA) and its composites are widely used in industrial applications; therefore, their durability is of great concern. In this study, the photooxidative degradation behavior of nanosilica-filled PMMA composite films and the cooperative effect of mixed solvents containing tetrahydrofuran (THF) and chloroform (TCM), as well as interfacial functional groups, was investigated. The surface functional groups of nanosilica fillers, such as polar, aryl, and alkyl moieties, significantly affect the photodegradation kinetics and pathways for PMMA. The key process lies in the modulation of solvent–solvent reaction selectivity at the polymer–filler interface. Functional groups that selectively promote the chlorination reaction between THF and TCM accelerate PMMA photodepolymerization, while those that suppress this reaction hinder degradation. This interfacial effect is validated by trends in molecular weight loss, volatile product profiles, and MMA yields during aging. Our findings reveal that the photodegradation behavior of PMMA composites is not only governed by environmental conditions but also critically influenced by interfacial chemistry. In this way, this study provides novel insight into the interfacial aging process for polymer nanocomposites, as well as guidance for the rational design of PMMA-based materials with improved durability.

## 1. Introduction

PMMA is a multifunctional amorphous polymer widely used in prosthodontics [1], automotive components [2,3], optical products [4,5] and coatings [6,7] because of its high transparency, impact resistance, and ease of processing. Since PMMA has the disadvantages of insufficient surface hardness, poor abrasion resistance, and low impact resistance, the performance of PMMA can be improved by adding inorganic fillers to prepare PMMA nanocomposites. Nanosilica (SiO_2_) was previously introduced into the PMMA matrix to improve the thermal stability [8], mechanical strength [9,10] and insulating properties [11] of the material due to the excellent physicochemical properties [12,13] induced by adding this filler. Chemically modifying the filler surface to form a good interfacial bond with the PMMA matrix is key to performance enhancement [14]. However, although the performance of PMMA composites can be sufficiently enhanced through the introduction of SiO_2_, the durability of these materials in service remains a critical issue that must be addressed. The sustained performance of such materials is closely related to various environmental factors, such as a combination of light, heat, oxygen, and humidity, which can lead to oxidization susceptibility and degradation during service, resulting in performance degradation and the inability to satisfy service requirements [15]. Consequently, to improve the durability of PMMA composites in long-term service, an understanding of its aging process is crucial.

Although the aging degradation process of PMMA itself [16,17,18] has been studied in detail, research on the aging process of PMMA composites remains limited. The effect of the alkyl chain length on the modified SiO_2_ surface on the thermal degradation behavior of PMMA has also been investigated [19]. The apparent thermal degradation activation energy for PMMA/SiO_2_ increases with an increase in the alkyl chain length on the surface of modified SiO_2_. The effects of other inorganic fillers, such as MMT [20,21], layered double hydroxides (LDHs) [22,23], TiO_2_ [24,25], Fe_2_O_3_ [21,26], Al_2_O_3_ [27,28], ZnO [29,30], Sb_2_O_3_ [31], ln_2_O_3_ [32], and CNTs [33,34], on the thermal stability of PMMA composites have also been extensively examined.

Most past studies focused on the effect of inorganic fillers on the thermal stability of PMMA composites. Conversely, studies on the photodegradation behavior of PMMA composites are rare. A visible light-triggered, main-chain-initiated depolymerization method capable of converting commercial PMMA into monomers with near-quantitative yields (>98%), regardless of the polymer’s molecular weight, end groups, or the presence of copolymers and additives, has been reported [35]. This approach relies on the in situ generation of chlorine radicals from chlorinated solvents such as 1,2-dichlorobenzene under violet LED irradiation. These radicals initiate hydrogen abstraction and β-scission reactions along the polymer mainchain. This method offers excellent tolerance to thermal history, multigram scalability, and recyclability, offering a promising strategy for the chemical recycling of commercial PMMA products such as Plexiglas.

Despite the impressive efficiency of this depolymerization method, it primarily addresses end-of-life recycling rather than the durability and degradation behavior of PMMA-based composites during service. The photooxidative aging process for PMMA nanocomposites under environmental stress conditions has also been examined. Including a residual tetrahydrofuran (THF) and chloroform (TCM) solvent mixture in PMMA films accelerates the photodegradation of PMMA, which involves the catalytic effect of solvent transformations [36]. However, in PMMA composites, the catalytic effect of solvent decomposition on PMMA photodegradation may be compromised due to interfacial interactions between filler surface functional groups and solvent molecules. Therefore, the photodegradation pathways for filled PMMA films containing residual solvents were systematically studied as a function of the surface functional groups introduced on nanosilica fillers. Unlike a previous report [35], which targeted post-consumer depolymerization via exogenous radical generation, the interfacial interactions and degradation chemistry occurring in situ during long-term exposure has been explored. This study may provide valuable insight into the design of durable PMMA-based materials.

In the present work, the surface of SiO_2_ was modified to contain polar functional groups, aryl groups, and alkyl groups. These modified SiO_2_ particles were then blended with PMMA in a solution to prepare composite film samples containing residual THF and TCM as mixed solvents. The changes to photodegradation products in the film samples during photooxidative aging were analyzed using the pyrolysis gas chromatography-mass spectrometry (Py-GC-MS) flash technique. This process enabled us to assess the influence of filler surface groups on the photodegradation behavior of SiO_2_-filled PMMA composite films containing residual mixed solvents. This analysis provides a better understanding of the interfacial interaction process during photodegradation. The results could be used to improve the durability design for PMMA and its composites and enhance the design of relevant photodegradation recycling strategies.

## 2. Materials and Methods

### 2.1. Materials and Preparation

In this work, commercially available atactic PMMA granules (IF850) were used and purchased from LG Chem, Seoul, Korea. The silica particles were hydrophilic fumed SiO_2_ (average particle size: 20 nm) purchased from Shanghai Aladdin Biochemical Technology Co. (Shanghai, China). The solvents, tetrahydrofuran (THF), and chloroform (TCM) used to prepare the solution casting PMMA films and the surfactant tetra-n-octylammonium bromide (TOAB) for the surface chemical modification of SiO_2_ were provided by Energy Chemical, Shanghai, China. The silane coupling agent and other reagents required for the surface chemical modification of SiO_2_ are described in Table 1.

**Figure 1:**
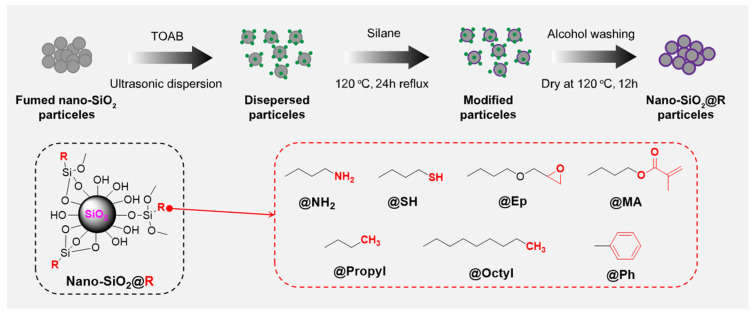
Schematic diagram of the surface chemical modification of SiO_2_.

The surface chemical modification process of SiO_2_ is shown in Figure 1. Firstly, 0.4 g of TOAB was dissolved in 75 mL of xylene solvent, to which 2.0 g of SiO_2_ particles was added and subjected to ultrasonic dispersion to obtain the dispersed particle suspension solution. The suspension was transferred to a 250 mL round-bottomed flask and placed in an oil bath at 120 °C. An excess silane coupling agent was slowly added dropwise to the flask, and the mixture was refluxed for 24 h. Specific dosing information of the silane coupling agent is shown in Table S1. After the reaction, five alcohol washes on the modified SiO_2_ were used to remove the surfactant and unreacted coupling agent and thus obtain a clean modified SiO_2_. Finally, the material was placed in a vacuum drying oven and dried at 120 °C for 12 h. From data presented in Table S1, SiO_2_-modified with surface polar functionalization {amino-modified (SiO_2_@NH_2_), epoxypropoxy-modified (SiO_2_@Ep), methacryloyloxy-modified (SiO_2_@MA), sulfhydryl-modified (SiO_2_@SH), surface arylation (SiO_2_@Ph) and surface alkylation [octyl-modified (SiO_2_@Propyl) and propyl-modified (SiO_2_@Octyl)]} were prepared, respectively.

SiO_2_-filled composite films with residual mixed solvents were prepared using the solution casting process. Firstly, 9.8 g of PMMA granules was weighed and dissolved in 40 mL of THF with magnetic stirring at room temperature until complete dissolution. Meanwhile, 0.2 g of SiO_2_@R (R stands for the different surface modification groups) particles was weighed, milled, and dispersed in 10 mL of TCM and sonicated for 30 min to obtain the dispersion, which was poured into the PMMA solution and magnetically stirred at room temperature for 30 min. Subsequently, the mixed solution was poured into a clean glass dish and evaporated for 48 h at room temperature under a fume hood. Finally, the obtained film samples were placed in a vacuum oven at 50 °C and held under a fixed load for 8 h, thereby obtaining SiO_2_-filled composite films in the presence of residual mixed solvents. As shown in Figure S1a in the Supplementary Material, the prepared PMMA/SiO_2_@R composite films contained approximately 15–18 wt% of residual mixed solvents before photooxidative aging (a weight loss temperature range of 50–250 °C was calculated). After photooxidative aging for 283 h, the residual mixed solvent content within the film decreased to approximately 10 wt%, as shown in Figure S1b. Figure S1 illustrates that even after being subjected to the vacuum oven and aging, the samples obtained by the solution cast film method still contained a certain amount of solvent. Thus, it was necessary to explore the cooperative aging of the solvent and the interface.

### 2.2. Photooxidative Aging

**Figure 2:**
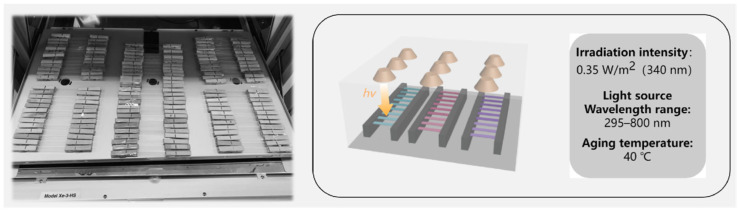
Photooxidative aging experiment: The left is a photo of the film in the aging chamber; the right is a schematic diagram.

The photooxidative aging experiments of the film samples were carried out in a xenon lamp aging chamber (Q-Sun Xe-3, Q-Lab Inc., Cleveland, OH, USA). Before the experiment, the films were cut into long strips of 100 mm × 15 mm, and the surface of each sample was cleaned. As shown in Figure 2, the ends of the samples were wrapped with tinfoil and fixed using clips, keeping the middle 50 mm of each sample exposed to light. Then, the samples were laid flat in the aging chamber. During photooxidative aging, the irradiation light source simulated the full sunlight spectrum with a wavelength range of 295–800 nm, an irradiation intensity of 0.35 W/m^2^ (340 nm), and an air temperature of 40 °C. The samples used for Py-GC-MS testing were taken from the light-exposed portion.

### 2.3. Characterization Methods

The volatile components in the photooxidative aging film samples were analyzed via a GCMS-QP2010SE gas chromatography-mass spectrometry (GC-MS) device manufactured by Shimadzu Corporation, Kyoto, Japan. An EGA/PY-3030D pyrolyzer (Frontier Lab, Fukushima, Japan) was equipped on the injection port, and the volatile components adsorbed in the film were collected using the flash vaporization technique. The test procedure was as follows. First, a sample of approximately 1.5 mg was weighed, the mass was recorded (accurate to 0.01 mg), and the sample was placed in the pyrolyzer. Then, the sample was flash evaporated at 300 °C for 30 s, and the volatile components obtained were loaded onto a column using a carrier gas (helium). The interface temperature was set to 250 °C. The Py-GC/MS test procedure was kept consistent for all samples to guarantee comparability of the test data, and the test was repeated at least three times. The specific peak area (SPA) was obtained by dividing the integrated area of the volatile component peaks in the total ion chromatogram (TIC) by the sample mass to reflect the yield of the volatile component in the sample after photooxidative aging, which was calculated with Equation (1):

(1) $$SPA=\frac{A_{voc}}{m}$$

where $A_{voc}$ is the integral area of the specific component peak in the total ion chromatogram of the volatile component adsorbed in the sample, and m is the sample mass in mg.

The functional groups on the surface of SiO_2_ particles before and after surface modification were characterized with Fourier transform infrared spectroscopy (FT-IR, IRTracer-100, SHIMADZU). The IR spectra were obtained by mixing the powdered samples with potassium bromide and pressing the samples into pellets during the testing process. In total, 32 scans were performed in transmission mode under a wave number range of 4000–400 cm^−1^ with a resolution of 4 cm^−1^.

The solvent content of the films was determined using a thermogravimetric analyzer (TGA, TG-209, NETZSCH, Selb, Germany) under a nitrogen atmosphere. The test procedure was as follows: First, approximately 5 mg of the film sample was weighed into an alumina crucible. Then, the temperature was ramped up from room temperature to 600 °C under a nitrogen atmosphere at a ramping rate of 10 °C/min. The amount of weight loss at 50–250 °C represented the amount of residual solvent in the sample. In addition, the thermogravimetric behavior of SiO_2_ particles before and after modification was analyzed. The test procedure was as follows: First, approximately 5 mg of the powder sample was placed into an alumina crucible. Then, the temperature was ramped up from room temperature to 100 °C and maintained for 5 min, before being ramped up to 900 °C at a ramping rate of 10 °C/min.

The ultraviolet-visible diffuse reflectance spectra (DRS) of SiO_2_ before and after surface chemical modification were analyzed using a UV-2600 spectrophotometer (Shimadzu, Kyoto, Japan) equipped with an integrating sphere accessory (ISR-2600Plus). The scanning wavelength range was set as 200–800 nm.

The chemical structure of the organic components grafted onto the surface of surface-modified SiO_2_ particles was analyzed via X-ray photoelectron spectroscopy (XPS, Escalab 250Xi, Thermo Fisher Scientific, Waltham, MA, USA). The peak splitting process of XPS photoelectron spectroscopy peaks was realized using the Avantage Software (powered by Thermo Fisher Scientific, V5.5), the data analysis software packaged with the spectrometer, and a binding energy of C_1s_, 284.8 eV was utilized to correctly charge the samples.

The molecular weight changes in the films before and after photooxidative aging were characterized with gel permeation chromatography (GPC, 1260 Infinity Ⅱ, Agilent Technologies, Santa Clara, CA, USA). Before testing, the films were fully dissolved in THF at a concentration of 10 mg/mL and filtered using a 25 μm pore size PTFE needle filter. During the test, the sample feed volume for each test was 20 μL, the liquid phase was THF, the flow rate of the unit pump was 1 mL/min, the temperature of the column chamber was 30 °C, and the column pressure was 38–40 bar. The molecular weights of all the samples were compiled for the efflux period from 7 min to 9.5 min.

Changes in the surface morphology of SiO_2_ before and after surface modification and the films before and after photooxidative aging were analyzed using a field emission scanning electron microscope (FE-SEM, JSM-7900F, JEOL, Tokyo, Japan). Prior to testing, the surface of the test samples was platinized for 120 s using a high vacuum ion sputtering apparatus (JFC-3000FC, JEOL, Japan). The accelerating voltage used during the test was 5.0 kV.

## 3. Results

### 3.1. Surface Structure Analysis of Modified SiO_2_

**Figure 3:**
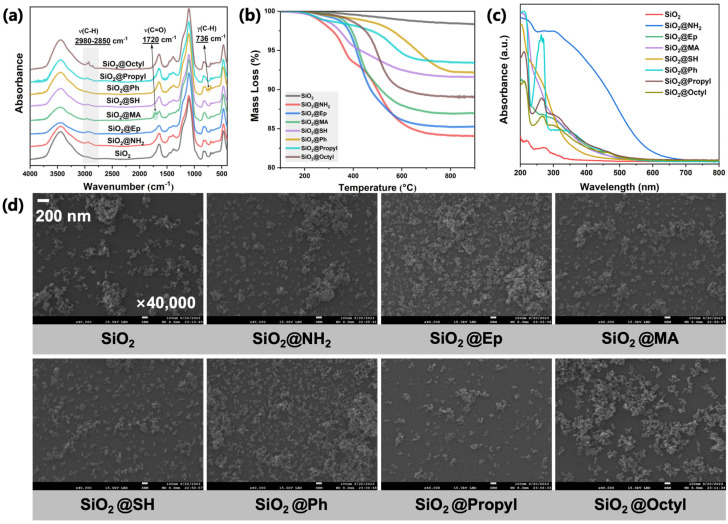
Surface structure analysis of SiO_2_ before and after surface modification: (**a**) FT-IR; (**b**) TGA; (**c**) UV-Vis; (**d**) SEM.

As shown in Figure 3a, new absorption peaks in the range of 2850–2980 cm^−1^ were observed in the FT-IR spectra of modified SiO_2_ particles compared with unmodified SiO_2_. These new peaks are attributed to the stretching vibration of the C-H bond. Although the absorption peak of the stretching vibration of the C-H bond of SiO_2_@Ph was not significant, a new absorption peak was observed at 736 cm^−1^, which is attributed to the out-of-plane bending vibration of the C-H bond on the benzene ring. In addition, an absorption peak from the stretching vibration of the C=O bond in the ester group was observed in the FT-IR spectrum of SiO_2_@MA. Based on the FT-IR results, it was preliminarily determined that the surfaces of all modified SiO_2_ particles contain specific organic components.

To further determine whether these organic components were covalently grafted onto the surfaces of SiO_2_ particles, the thermogravimetric behavior of the organic components on the surfaces of modified SiO_2_ particles was analyzed via TGA. As shown in Figure 3b, compared to unmodified SiO_2_ particles, all modified SiO_2_ particles exhibited greater mass loss due to the presence of organic components on their surfaces, with the temperature range of mass loss occurring in the range of 300–800 °C. Considering that the boiling points of the silane coupling agents used for the chemical modification of the surfaces are within 300 °C (see Table 1), we determined that the extra mass loss of the modified SiO_2_ particles was contributed by the covalently grafted silane coupling agents on the surfaces and not by residual coupling agents physically adsorbed on the surfaces.

The effects of surface modification on the absorption and morphological properties of SiO_2_ particles were analyzed via UV-Vis and SEM. As shown in Figure 3c, all SiO_2_ particles after surface modification exhibited significantly enhanced ultraviolet and visible light absorption capabilities. As shown in Figure 3d, the SEM photos of SiO_2_ before and after surface modification showed that the modified nano-SiO2 had similar particle sizes, albeit with some aggregation.

**Figure 4:**
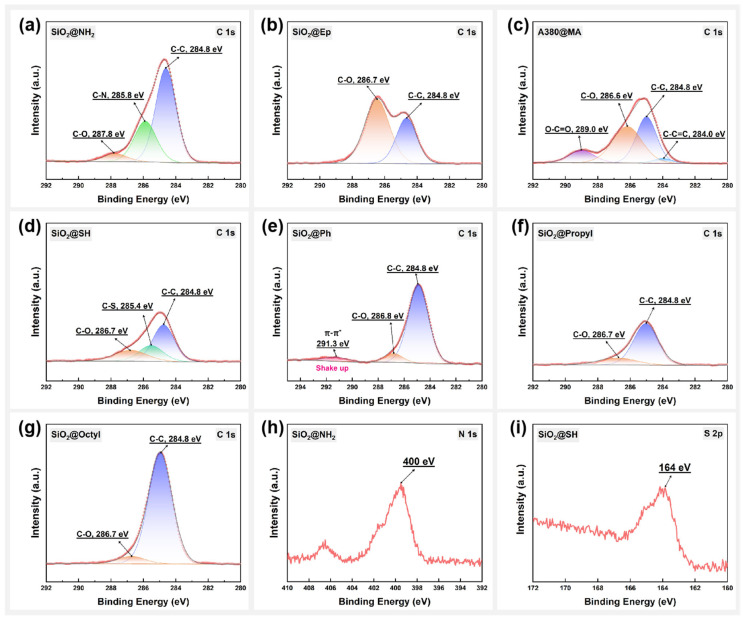
X-ray photoelectron spectra (XPS) of surface-modified SiO_2_ particles: (**a**–**g**) C_1s_ orbital peaks; (**h**) N_1s_ orbital peaks; (**i**) S_2p_ orbital peaks.

The chemical structure of the surface composition of modified SiO_2_ particles was further analyzed using XPS. Figure 4a–g presents the C_1s_ orbital energy spectral signals of the organic components on the surfaces of seven types of surface-modified SiO_2_ particles, showing significant differences in the chemical state in which the C atoms of the organic components on the surface of the SiO_2_ particles were found in the different surface-modified SiO_2_ samples. By splitting the C_1s_ peaks, the different chemical states of the C atoms in the organic components on the surface were determined. As shown in Figure 4a, the C_1s_ energy spectrum of SiO_2_@NH_2_ particles consisted of three peaks, with the C-C peak at 284.8 eV. At the same time, the electronegativity of the surrounding atoms affected the significant chemical shift in the inner-shell electrons of C-atoms to high binding energy. Figure 4h shows the presence of the N_1S_ signal peaks (400 eV) in the organic components on the surfaces of the SiO_2_@NH_2_ particles. Therefore, we determined that the peak at 285.8 eV can be attributed to C-N. We also observed a very small peak at 287.8 eV, which may be attributable to C-O based on the electronegativity of the atoms (C < N < O). The C_1s_ spectral peaks of the organic components on the surfaces of the SiO_2_@Ep particles consisted of two peaks: C-C (284.6 eV) and C-O (286.8 eV). Due to the large proportion of C-O structures in the organic components on the surfaces of SiO_2_@Ep particles, the C-O peak was observed to be particularly significant in its C_1s_ energy spectrum, where the peak area exceeded that of the C-C peak (Figure 4b). As shown in Figure 4c, the C-C peak (284.8 eV), C-O peak (286.6 eV), C-C=C peak (284.0 eV), and O-C=O peak (289.0 eV) are observable in the C_1s_ spectrum of the SiO_2_@MA particles, which is in perfect agreement with the chemical structure of their terminal groups. The peak at 285.4 eV in the C_1s_ spectrum of SiO_2_@SH particles in Figure 4d is attributed to the C-S bond. The photoelectron signals from the S_2p_ orbitals in the sulfhydryl group can also be observed in Figure 4i. In the C_1s_ energy spectrum of SiO_2_@Ph particles, a shake up peak appeared at 291.3 eV, as shown in Figure 4e. This peak represents a characteristic companion peak with a discrete appearance on the low kinetic energy side in the benzene ring. Figure 4f,g present the C_1s_ energy spectrum of two types of acetylation-modified SiO_2_ particles, with C-C peaks dominating the structure. In addition, it can be observed that the C-C peaks in the C_1s_ energy spectra of the SiO_2_@Octyl particles are larger than those of the SiO_2_@Propyl particles, mainly due to the longer alkyl chains on the surfaces. Based on the XPS analysis, all modified SiO_2_ particles were successfully covalently grafted with specific functional groups on their surfaces.

### 3.2. Analysis of Photodegradation Products

**Figure 5:**
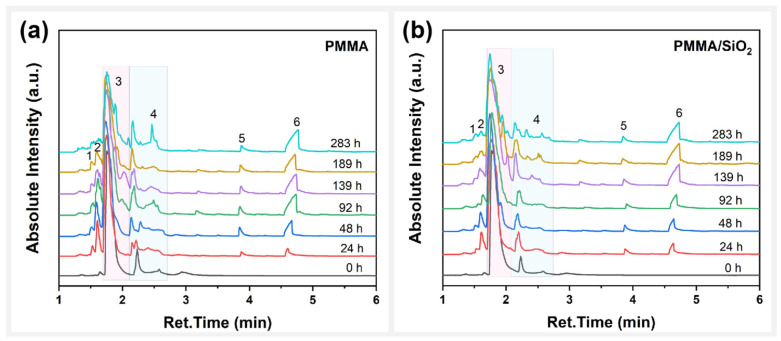
Total ion chromatogram (TIC) of the samples with photooxidative aging time: (**a**) PMMA film; (**b**) PMMA/SiO2 film.

**Figure 6:**
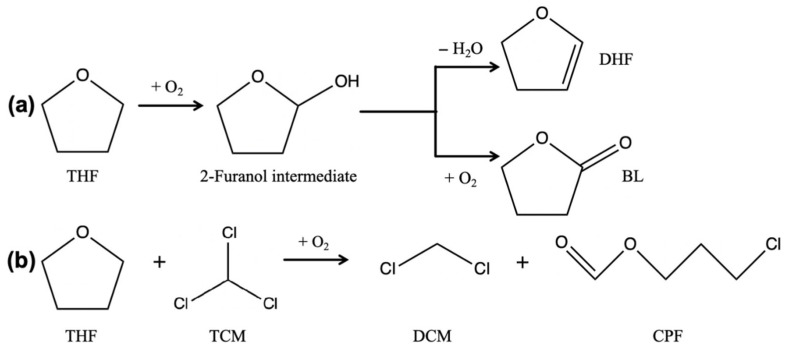
Two types of THF reactions: (**a**) oxidation reaction of itself; (**b**) reaction with TCM.

The TICs of PMMA and PMMA/SiO_2_ films at different photooxidative aging times are presented in Figure 5a and b, respectively. The volatile components generated from the photodegradation of PMMA are shown in Table 2. It can be observed that both samples have the same volatile components consisting of dichloromethane (DCM, peak 1), 2,3-dihydrofuran (DHF, peak 2), methyl methacrylate (MMA, peak 4), 3-chloropropyl formate (CPF, peak 5), and γ-butyrolactone (BL, peak 6). MMA was derived from the photodepolymerization process of PMMA. Here, the accumulation of MMA during photooxidative aging reflects the speed of the photodepolymerization reaction. DCM, DHF, CPF, and BL were all produced from the reactions between residual solvents during photodegradation of the composite films. Among them, THF underwent two reactions: The first was a reaction with TCM to produce the chlorine-containing products DCM and CPF. The second was the oxidation reaction of THF itself to produce the oxidation products DHF and BL, which are presented in Figure 6a and b, respectively.

To determine whether the yield of MMA during PMMA photodegradation is affected by the reactions of residual solvents within the composite films, the yields of the two types of solvent reaction products during the photodegradation process were calculated using Equations (2) and (3):

(2) $${SPA}_{THF+O_{2}}=\frac{A_{DHF}+A_{BL}}{m}$$

(3) $${SPA}_{THF+TCM}=\frac{A_{DCM}+A_{CPF}}{m}$$

where ${SPA}_{THF+O_{2}}$ is the total specific peak area of DHF and B; ${SPA}_{THF+TCM}$ is the total specific peak areas of DCM and CPF; $A_{DHF}$, $A_{BL}$, $A_{DCM}$, and $A_{CPF}$ are the integral areas of the peaks corresponding to the components of the photodegradation products, respectively; and m is the mass of the sample in mg.

**Figure 7:**
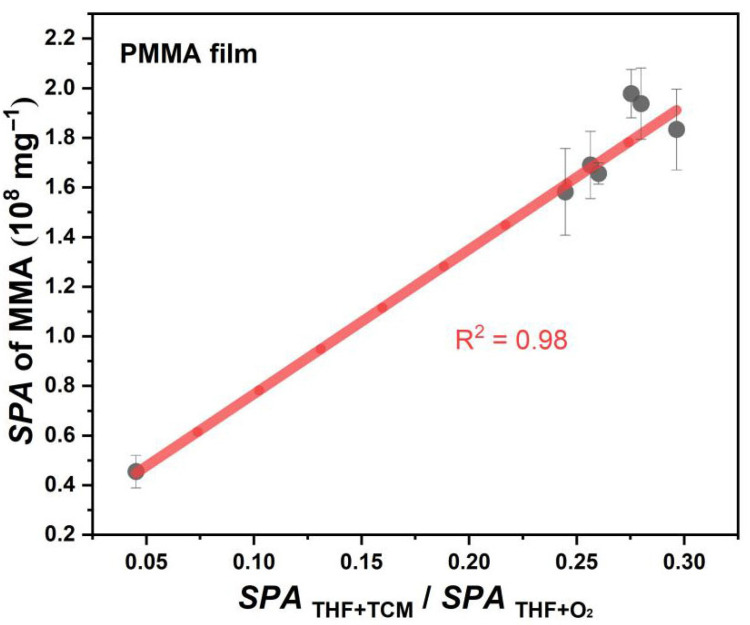
Relationship between the yield of MMA during PMMA photodegradation and the reactions of the two types of solvents.

As shown in Figure 7, the yield of MMA is positively correlated with ${SPA}_{THF+TCM}/{SPA}_{THF+O_{2}}$ during the photodegradation of PMMA film, which means that the solvent reactions will affect the photodegradation process of PMMA. During photodegradation, the more THF in the PMMA film tends to react with TCM, the more likely it is to promote the generation of MMA. This promotion mainly occurs because an increase in the selectivity of the reaction between THF and TCM will be more conducive to promoting the chlorination of ester groups on the PMMA side chain, thereby accelerating the photodepolymerization of PMMA.

### 3.3. Effect of Surface Polar Functionalization Modification on the Photodegradation Behavior of PMMA/SiO_2_

**Figure 8:**
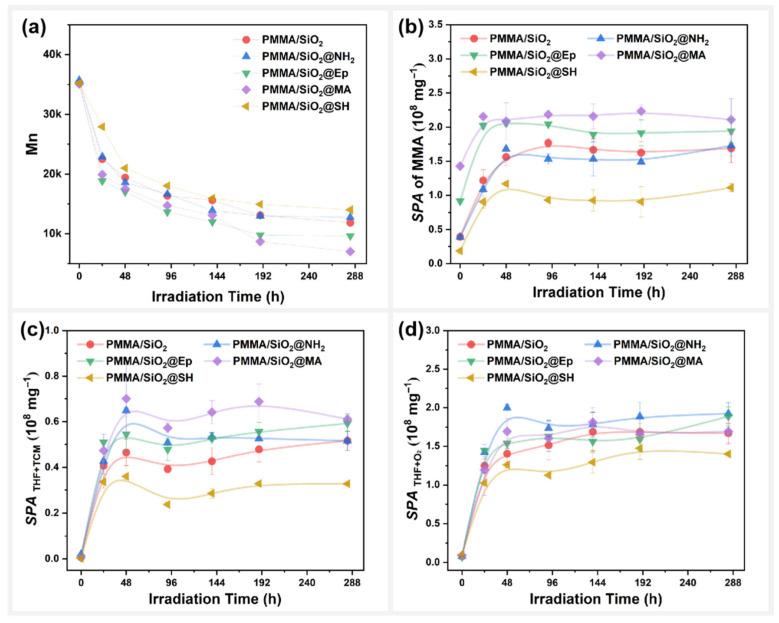
PMMA/SiO_2_ composite films with different polar modifications on the surface: variation in (**a**) molecular weight (Mn), (**b**) specific peak area of MMA, (**c**) total specific peak area of DCM and CPF, and (**d**) total specific peak area of DHF and BL with photooxidative aging time.

Changes in the molecular weight (Mn) of the PMMA/SiO_2_ composite films during photooxidative aging were also compared, as shown in Figure 8a. The results show that modifying the SiO_2_ surface with different polar functional groups will significantly change the photodegradation behavior of PMMA films. The decrease in the Mn content of the composite films, from high to low, was as follows: PMMA/SiO_2_@MA > PMMA/SiO_2_@Ep > PMMA/SiO_2_@NH_2_ ≈ PMMA/SiO_2_ > PMMA/SiO_2_@SH. Clearly, amino group modification (@NH_2_) on the surface of SiO_2_ did not affect the degree of photodegradation of PMMA composite films. Both epoxy group modification (@Ep) and methacryloyloxy group modification (@MA) promoted the photodegradation of PMMA composite films. However, sulfhydryl group modification (@SH) inhibited the photodegradation of PMMA composite films. The surface morphology changes in PMMA/SiO_2_@SH were, therefore, minimal, as shown in Figure S2.

Figure 8b shows changes to the MMA specific peak area (SPA) in the PMMA composite films during photooxidative aging. The photodepolymerization speed of the PMMA composite films from high to low was as follows: PMMA/SiO_2_@MA > PMMA/SiO_2_@Ep > PMMA/SiO_2_ @NH_2_ ≈ PMMA/SiO_2_ > PMMA/SiO_2_@SH. This result is consistent with the molecular weight of the PMMA composite films, further confirming that polar functional group modification on the SiO_2_ surface will affect the photodegradation behavior of PMMA composite films.

We also sought to determine whether the reaction between the residual mixed solvents will be affected by different polar functional groups on the SiO_2_ surface during the photodegradation of PMMA composite films. Figure 8c and 8d, respectively, present the changes in the products of the reaction between THF and TCM, as well as the products of the oxidation reaction of THF during photooxidative aging. For ${SPA}_{THF+TCM}$, the effects of polar functional group modifications on the SiO_2_ surface on the reaction activity of THF with TCM were as follows, from high to low: SiO_2_@MA > SiO_2_@Ep ≈ SiO_2_@NH_2_ > SiO_2_ > SiO_2_@SH. For ${SPA}_{THF+O_{2}}$, the effects of polar functional group modifications on the SiO_2_ surface on the oxidation activity of THF itself were as follows, from high to low: SiO_2_@NH_2_ > SiO_2_ ≈ SiO_2_@MA ≈ SiO_2_@Ep > SiO_2_@SH. Unlike unmodified SiO_2_, SiO_2_@MA and SiO_2_@Ep were able to promote the reaction between THF and TCM and had little effect on the oxidation reaction of THF itself. SiO_2_@NH_2_ was able to promote both solvent reactions, whereas SiO_2_@SH instead inhibited the solvent reactions.

Apparently, the photodegradation of PMMA composite films was accelerated when the THF reaction with TCM was promoted, and the oxidation reaction of THF itself was not affected. When the reaction of THF with TCM and the THF oxidation reaction were promoted simultaneously, the photodegradation of PMMA composite films was not affected. When the reaction of THF with TCM was inhibited, the photodegradation of the PMMA composite film was delayed. Therefore, the effect of modifying polar functional groups on the filler surface on solvent reactivity was the main reason for the changes observed in the photodegradation behavior of the PMMA composite films.

### 3.4. Effect of Surface Arylation Modification on the Photodegradation Behavior of PMMA/SiO_2_

**Figure 9:**
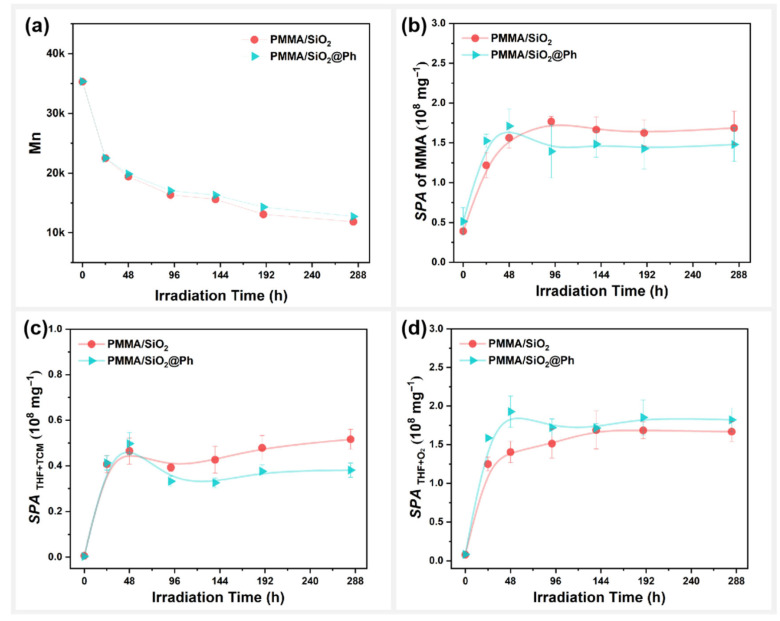
PMMA/SiO_2_ composite films with surface arylation modifications: variation in the (**a**) molecular weight (Mn), (**b**) specific peak area of MMA, (**c**) total specific peak area of DCM and CPF, and (**d**) total specific peak area of DHF and BL with photooxidative aging time.

Figure 9 presents the photodegradation behavior of the SiO_2_-filled PMMA composite films before and after surface arylation modification. Here, the molecular weight decrease in PMMA composite films with surface arylation modification was always slightly higher than that without modification during photooxidative aging (Figure 9a). Similar results were observed for the specific peak area (SPA) of MMA during photooxidative aging (Figure 9b). Although the photodepolymerization speeds of the PMMA composite films before and after modification were comparable at the early stage of aging, the photodepolymerization speed of the PMMA/SiO_2_@Ph films was consistently higher than that of the PMMA/SiO_2_ films thereafter. These results indicate that arylation modification on the surface of SiO_2_ inhibited the photodegradation of PMMA composite films. The surface morphology changes in PMMA/SiO_2_@Ph films were, therefore, much less significant than those of the PMMA/SiO_2_ films, as presented in Figure S2.

For ${SPA}_{THF+TCM}$ and ${SPA}_{THF+O_{2}}$, as shown in Figure 9c,d, the reactivity of THF with TCM in the photodegradation of PMMA composite films decreased after arylation modification on the surface of SiO_2_, whereas the reactivity of THF oxidation increased. This result indicates that a decrease in the reactivity of THF with TCM remaining in the PMMA composite film during photodegradation will certainly lead to a slower photodegradation speed for PMMA. This phenomenon mainly occurs because the chlorination of ester groups in PMMA photodepolymerization is limited by TCM reactivity.

### 3.5. Effect of Surface Alkylation Modification on the Photodegradation Behavior of PMMA/SiO_2_

**Figure 10:**
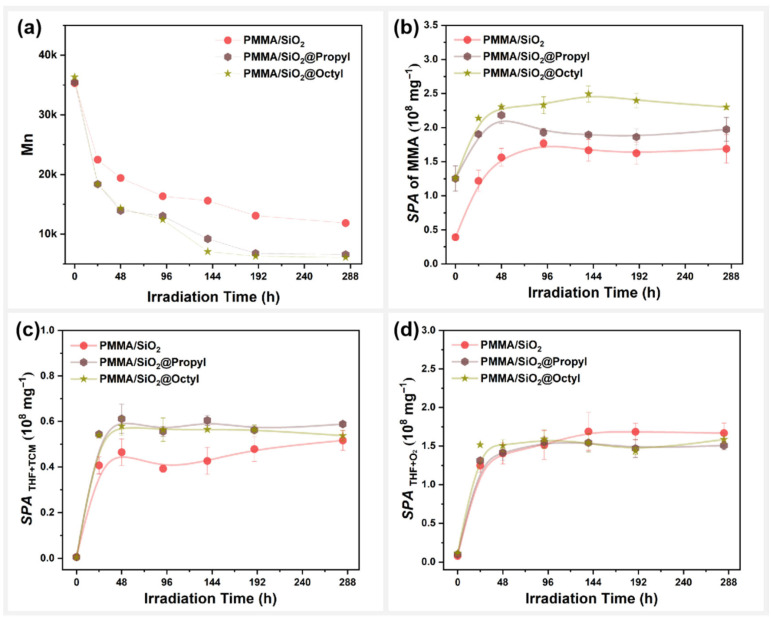
PMMA /SiO_2_ composite films with surface alkylation modification: variations in the (**a**) molecular weight (Mn), (**b**) specific peak area of MMA, (**c**) total specific peak area of DCM and CPF, and (**d**) total specific peak area of DHF and BL with photooxidative aging time.

The photodegradation behavior of SiO_2_-filled PMMA composite films before and after different alkylation surface modifications is shown in Figure 10. Here, both alkylation modifications on the surface of SiO_2_ lead to a significantly higher molecular weight decrease for the PMMA composite films during photooxidative aging (Figure 10a). Similar results were obtained in the changes in specific peak area of MMA (SPA) during photooxidative aging (Figure 10b). These results indicate that the alkylation modification on the surface of SiO_2_ promoted the photodegradation of PMMA composite films, possibly because the longer alkyl chains on the surface of SiO_2_@Octyl are more favorable to the photothermal effect, thereby accelerating the photodepolymerization of PMMA.

Figure 10c,d present changes in the reaction products between THF and TCM, as well as the products of the oxidation reaction of THF for PMMA composite films with alkylation modifications during photooxidative aging. For ${SPA}_{THF+TCM}$ and ${SPA}_{THF+O_{2}}$, the reactivity of THF with TCM in the photodegradation of PMMA composite films increased after alkylation modification on the surface of SiO_2_, whereas the oxidation reactivity of THF itself was not affected. The photodegradation behaviors of PMMA composite films were the same as those of SiO_2_@MA and SiO_2_@Ep. Therefore, the photodegradation behaviors of the composite films were similar, as they all exhibited accelerated PMMA photodegradation.

### 3.6. Interfacial Effect in Photodegradation

**Figure 11:**
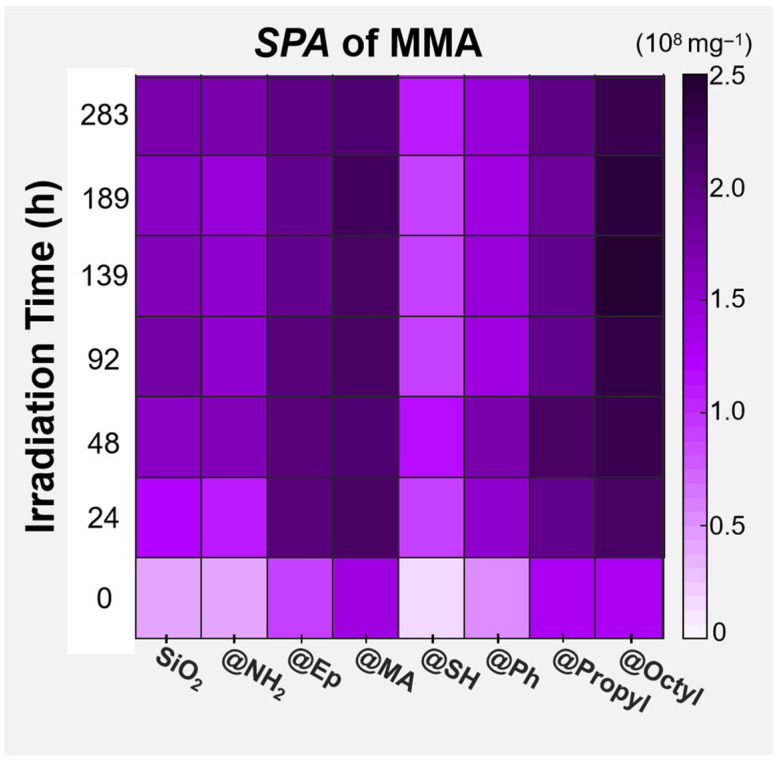
Comparative hotspot map of changes in the specific peak area of MMA for the PMMA composite films filled with SiO_2_ modified using different surface chemical modifications during photooxidative aging.

Changes in the MMA yield of PMMA/SiO_2_ composite films with different surface chemical modifications during photooxidative aging were compared using a hotspot map, as shown in Figure 11. It can be seen that the effects of different surface groups on the photodepolymerization rate of the PMMA composite films were as follows, from high to low:

@Octyl > @MA > @Propyl ≈ @Ep > @NH_2_ ≈ @SiO_2_ > @Ph > @SH.

**Figure 12:**
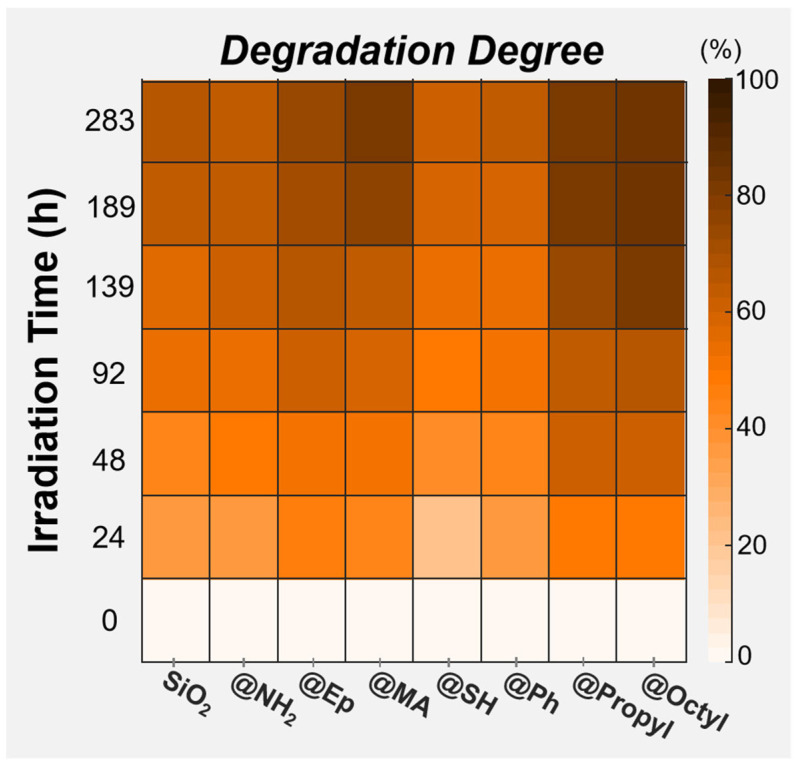
Comparative hotspot map of changes in the degradation degree (DD) for PMMA composite films filled with SiO_2_ modified by different surface chemical modifications during photooxidative aging.

To evaluate the effects of different surface groups of SiO_2_ on the photodegradation ability of PMMA composite films, the photodegradation degree (DD) of different composite films during photooxidative aging was also compared using a hotspot map, as shown in Figure 12. The calculation formula for DD is as follows:

(4) $$DD=\frac{{Mn}_{0}-{Mn}_{t}}{{Mn}_{0}}\times 100\%$$

where ${Mn}_{0}$ is the number average molecular weight of the sample before photooxidative aging, and ${Mn}_{t}$ is the number average molecular weight of the sample after t hours of photooxidative aging.

According to the DD changes, the effects of different surface groups of SiO_2_ on the photodegradation ability of the PMMA composite films were as follows, from high to low: @Octyl ≈ @Propyl ≈ @MA > @Ep > @NH_2_ ≈ SiO_2_ > @Ph ≈ @SH.

Changes in the SPA of MMA and those of DD were affected by the surface groups of SiO_2_ in almost the same way, which again indicates that the groups on the surface of the filler are the key factor influencing the photodegradation behavior of PMMA composite films. This phenomenon is related to the selective promotion or inhibition of surface groups on the reaction of THF with TCM and the oxidation reaction of THF itself. Next, a solvent reaction selectivity index (SI) was used to more clearly compare the trends of these two solvent reactions during the photodegradation of different PMMA composite films. The ${SI}_{t}$ of the sample after t hours of photooxidative aging was calculated as follows:

(5) $${SI}_{t}=\frac{{SPA}_{THF+TCM}^{t}}{{SPA}_{THF+O_{2}}^{t}}$$

where ${SPA}_{THF+TCM}^{t}$ is the total specific peak areas of DCM and CPF in the sample after t hours of photooxidative aging, and ${SPA}_{THF+O_{2}}^{t}$ is the total specific peak areas of DHF and CPF in the sample after t hours of photooxidative aging. The SI of a sample is the statistical mean value, where a higher value represents a greater tendency to promote the reactivity of THF with TCM.

**Figure 13:**
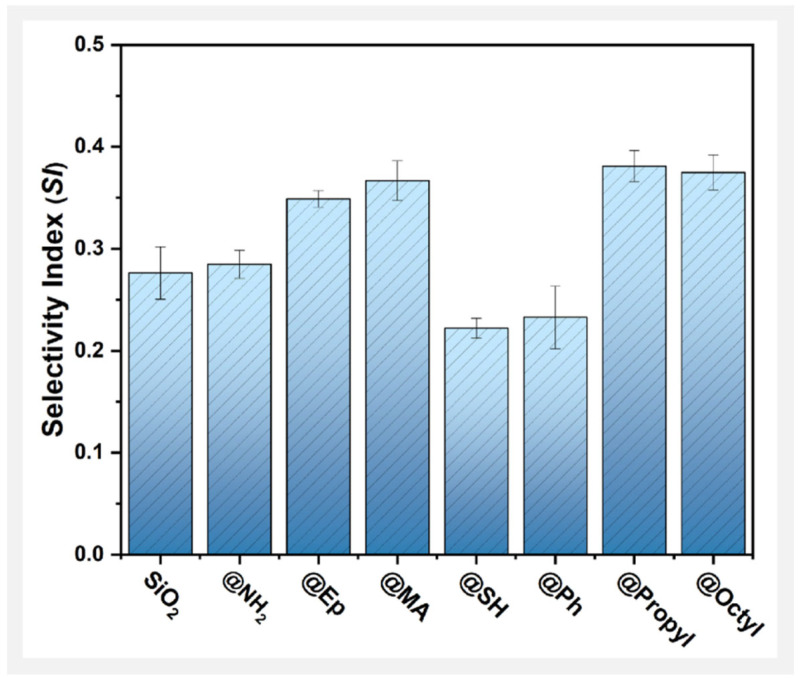
Selectivity index (SI) of solvent reactions in different SiO_2_-filled PMMA composite films.

The SI curves of different PMMA composite films are compared in Figure 13. Here, the effects of different groups on the surface of SiO_2_ on the SI were as follows, from high to low: @Propyl ≈ @Octyl ≈ @MA > @Ep > @NH_2_ ≈ SiO_2_ > @Ph ≈ @SH. This result coincides with the changes in the DD of the PMMA composite films. Therefore, the higher the reactivity of THF with TCM in the photodegradation of the PMMA/SiO_2_ composite films, the stronger the photodegradation ability.

Table 3 summarizes the effects of different groups on the surface of SiO_2_ on the parameters related to the photodegradation of the PMMA composite films. The octyl-, propyl-, MA-, and Ep- groups on the surface of SiO_2_ all selectively promoted the reaction of THF with TCM. The SH- and Ph- groups on the surface of SiO_2_ both selectively inhibited the reaction of THF with TCM. Conversely, the NH_2_- groups on the surface of SiO_2_ did not selectively contribute to the reaction of THF with TCM. Additionally, the presence of surface groups affected the reactivity of solvent molecules at the interface. Ultimately, differences in the interactions between different surface groups and the solvent molecules at the interface led to the selectivity of the solvent reaction.

**Figure 14:**
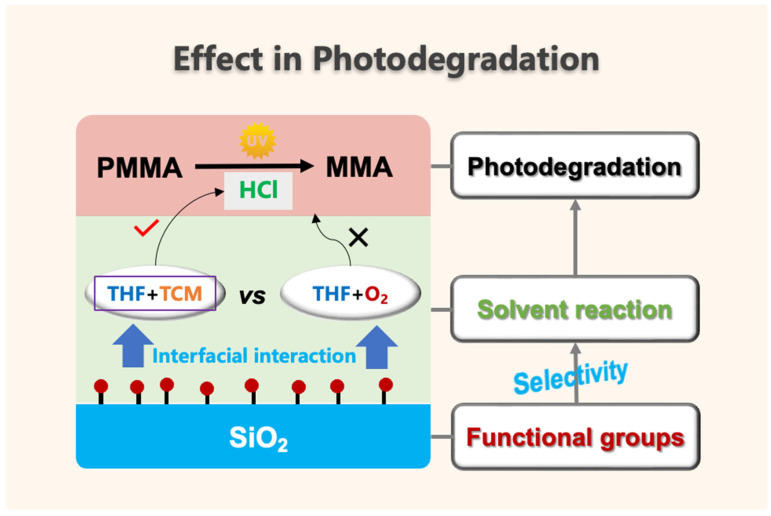
Effect of interfacial interaction in the photodegradation of PMMA/SiO_2_ composite films.

The interfacial effect of PMMA/SiO_2_ composite films in photodegradation is summarized in Figure 14. The interactions between the functional groups on the SiO_2_ surface and the solvent molecules at the interface altered the reactivity between the solvent molecules. As a result, the surface groups selectively promoted or inhibited the reaction of THF with TCM and the oxidation reaction of THF itself, ultimately changing the photodegradation behavior of the PMMA composite films. The photodegradation of the PMMA composite films was accelerated only when the groups on the surface of the SiO_2_ exerted a selective promotion effect on the reaction between THF and TCM.

## 4. Conclusions

In this study, the photodegradation behavior of a series of PMMA/SiO_2_ composite films was analyzed. Additionally, the effects of surface functional groups of SiO_2_ and their interactions with mixed solvents containing THF and TCM were described.

(1) Polar modification, arylation modification, and alkylation modification on the surface of SiO_2_ significantly affected the photodegradation behavior of the SiO_2_-filled PMMA composite films with residual mixed solvents. Among them, @Octyl, @Propyl, @MA, and @Ep modifications promoted the photodegradation of the PMMA composite films; @SH and @Ph modifications inhibited the photodegradation of the PMMA composite films; and @NH_2_ modifications had no effect on the photodegradation rate of the PMMA composite films.
(2) The effects of surface groups on SiO_2_ on solvent reactivity were the main reason for the different photodegradation behavior of PMMA composite films with residual mixed solvents.
(3) We proposed a cooperative effect in the photodegradation of PMMA/SiO_2_ composite films with residual mixed solvents. The photodegradation of PMMA composite films was accelerated only when the surface group of SiO_2_ exhibited a selective promotion effect on the reaction between THF and TCM.

## Figures and Tables

**Table 1:** Information on reagents used in the chemical modification of SiO_2_ surfaces.

Reagents	Features	Abbreviations	Company
Xylene	AR, ≥99.7%		Greagent, Shanghai, China
Anhydrous ethanol	AR, ≥99.7%		Greagent, Shanghai, China
Octyltrimethoxysilane	Purity: 98% ρ = 0.91 g/mL Boiling point: 191.5 ± 8.0 °C	OTMS	Heowns Biochem Technologies. Llc. Tianjin, China
Propyltriethoxysilane	Purity: 98% ρ = 0.89 g/mL Boiling point: 168.4 ± 8.0 °C	PTES	Bide Pharmatech Co., Ltd. Shanghai, China
γ-(2,3-epoxypropoxy) propyltrimethoxysilane	Purity: 98% ρ = 1.07 g/mL Boiling point: 299.4 °C	EPPTMS	Energy Chemical, Anhui Senrise Technologies Co., Ltd., Hefei, China
3-mercaptopropyltri- methoxysilane	Purity: 98% ρ = 1.06 g/mL Boiling point: 198.0 °C	MPTMS	Energy Chemical, Anhui Senrise Technologies Co., Ltd.
3-aminopropyltriethoxy-silane	Purity: 99.5% ρ = 0.95 g/mL Boiling point: 222.1 ± 13.0 °C	APTES	Energy Chemical, Anhui Senrise Technologies Co., Ltd.
γ-methacryloxypropyltri-methoxysilane	Purity: 98% ρ = 1.04 g/mL Boiling point: 190 °C	MAPTMS	Shanghai Dibai Biotechnology Co., Ltd., Shanghai, China
Phenyltrimethoxysilane	Purity: 99.5% ρ = 1.06 g/mL Boiling point: 185.7 ± 9.0 °C	PhTMS	Energy Chemical, Anhui Senrise Technologies Co., Ltd.

**Table 2:** Identification of volatile components generated during the photooxidative aging of PMMA and PMMA/SiO_2_ films; labels in the table correspond to Figure 5**.**

Peaks	Ret. Time	Volatile Products	Molecular Structure
1	1.6	Dichloromethane (DCM)	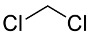
2	1.7	2,3-dihydrofuran (DHF)	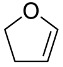
3	1.7–2.2	Solvents	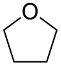 or 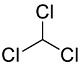
4	2.2–2.8	Methyl methacrylate (MMA)	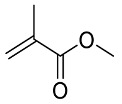
5	4.2	3-Chloropropyl formate (CPF)	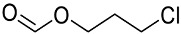
6	4.8	γ-butyrolactone (BL)	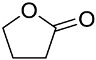

**Table 3:** Effects of different groups on the surface of SiO_2_ on the parameters related to the photodegradation of PMMA composite films under residual mixed solvents, with reference to the PMMA/SiO_2_ film.

Modified SiO_2_	${SPA}_{THF+TCM}$		
SiO_2_@NH_2_	**↑**	**↑**	**-**
SiO_2_@Ep	**↑**	**-**	**↑**
SiO_2_@MA	**↑↑**	**-**	**↑↑**
SiO_2_@SH	**↓↓**	**↓**	**↓**
SiO_2_@Ph	**↓**	**↑**	**↓**
SiO_2_@Propyl	**↑↑**	**-**	**↑**
SiO_2_@Octyl	**↑↑**	**-**	**↑↑↑**

$\;{SPA}_{THF+TCM}$ reflects the reactivity of THF with TCM; ${SPA}_{THF+O_{2}}$ reflects the photooxidative reactivity of THF itself; **↑** represents promotion, **↓** represents inhibition, where a higher quantity corresponds to a stronger effect; - represents no effect.

## Data Availability

Data are contained within the article.

## Supplementary Materials

The following supporting information can be downloaded at https://www.mdpi.com/article/10.3390/polym17162241/s1, Figure S1: Thermogravimetric curves of PMMA and its SiO_2_ filled composite films before and after photooxidative aging; Figure S2: Surface morphology changes of the film during photooxidative aging; Table S1 Feeding information of silane coupling agents for SiO2 surfaces chemical modification. Reference [37] are cited in the supplementary materials.
