# Supplementary material for: Photodegradation Behavior of Nanosilica-Filled PMMA Composite: Cooperative Effect of Mixed Solvents and Interfacial Functional Groups
Source: Polymers (Basel). 2025 Aug 19;17(16):2241. doi: 10.3390/polym17162241 (PMC12390198; doi:10.3390/polym17162241)
Supplement: Supplementary file 1 [file polymers-17-02241-s001.zip › polymers-3800840-supplementary.pdf]

Photodegradation behavior of nanosilica-filled PMMA composite: synergistic effect of mixed solvents and interfacial functional groups

Supplementary Material

Zhiping Xu[1†], Liangchen Li[1†], Ying Liu[1], Rui Yang[1*]

1. Department of Chemical Engineering, Tsinghua University, Beijing, China

*Corresponding author(s), E-mail(s): yangr@mail.tsinghua.edu.cn

†These authors contributed equally to this work

Table S1 lists the specific feeding information of Nano-SiO_2 with different surface chemical modifications. According to the literature, the maximum density of hydroxyl groups that can be accommodated on the surface of silica is about 5 hydroxyl groups/nm [36]. Therefore, in this work, the required silane coupling agent additions were all calculated based on the molar ratio of surface silica hydroxyl groups to coupling agent molecules of 1:4.

Table S1 Feeding information of silane coupling agents for SiO_2 surfaces chemical modification

Silanes	Feed	Products
APTES	12.6 mmol (2.95 ml)	SiO_2@NH_2
EPPTMS	12.6 mmol (2.78ml)	SiO_2@Ep
MAPTMS	12.6 mmol (2.84 ml)	SiO_2@MA
MPTMS	12.6 mmol (2.34 ml)	SiO_2@SH
PhTMS	12.6 mmol (2.36 ml)	SiO_2@Ph
PTES	12.6 mmol (2.91 ml)	SiO_2@Propyl
OTMS	12.6 mmo (3.26 ml)	SiO_2@Octyl

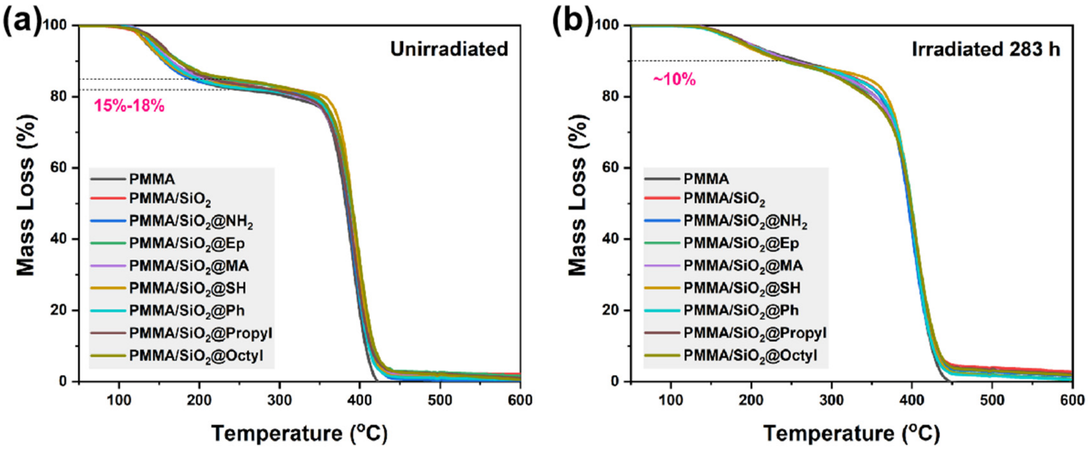

Fig. S1 Thermogravimetric curves of PMMA and its SiO_2 filled composite films before and after photooxidative aging: **(a)** before photooxidative aging; **(b)** after photooxidative aging for 283h

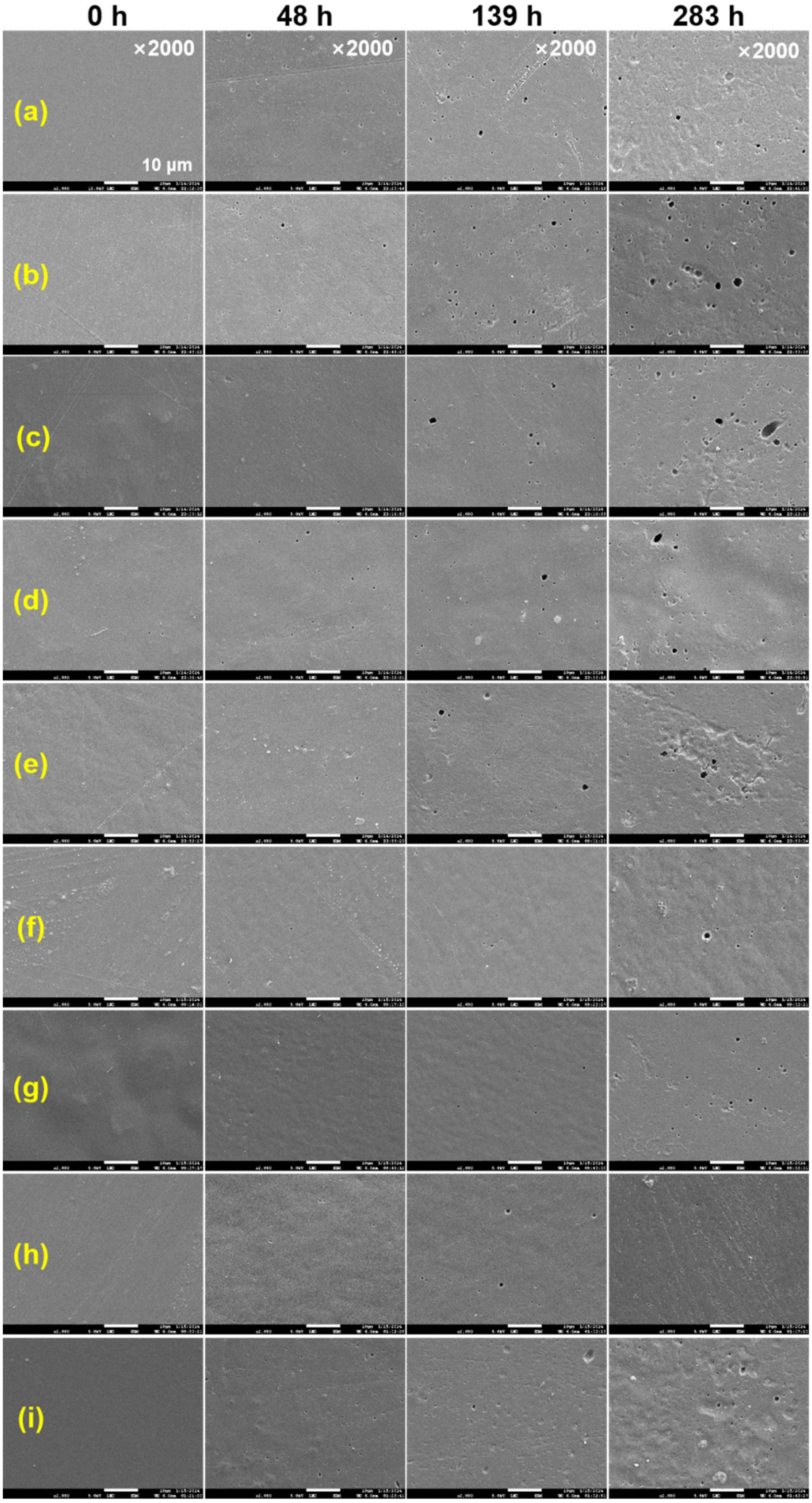

Fig. S2 Surface morphology changes of the film during photooxidative aging: **(a)** PMMA; **(b)** PMMA/SiO_2; **(c)** PMMA/SiO_2@NH_2; **(d)** PMMA/SiO_2@Ep; **(e)**

PMMA/SiO_2@MA; **(f)** PMMA/SiO_2@SH; **(g)** PMMA/SiO_2@Ph; **(h)** PMMA/SiO_2@Propyl; **(i)** PMMA/SiO_2@Octyl

References

37. Zhuravlev LT (2000) The surface chemistry of amorphous silica. Zhuravlev model. *Colloids Surf Physicochem Eng Asp* 173(1):1-38. https://doi.org/10.1016/S0927-7757(00)00556-2
